# CVDF DYNAMIC—A Dynamic Fuzzy Testing Sample Generation Framework Based on BI-LSTM and Genetic Algorithm

**DOI:** 10.3390/s22031265

**Published:** 2022-02-07

**Authors:** Mingrui Ma, Lansheng Han, Yekui Qian

**Affiliations:** 1 School of Cyber Science and Engineering, Huazhong University of Science and Technology, Wuhan 430074, China; jkpathfinder@hust.edu.cn; 2 PLA Army Academy of Artillery and Air Defense, Zhengzhou 450052, China; scienceart2021@163.com

**Keywords:** genetic algorithm, Bi-LSTM neural network, fuzzy testing sample generation, deep learning

## Abstract

As one of the most effective methods of vulnerability mining, fuzzy testing has scalability and complex path detection ability. Fuzzy testing sample generation is the key step of fuzzy testing, and the quality of sample directly determines the vulnerability mining ability of fuzzy tester. At present, the known sample generation methods focus on code coverage or seed mutation under a critical execution path, so it is difficult to take both into account. Therefore, based on the idea of ensemble learning in artificial intelligence, we propose a fuzzy testing sample generation framework named CVDF DYNAMIC, which is based on genetic algorithm and BI-LSTM neural network. The main purpose of CVDF DYNAMIC is to generate fuzzy testing samples with both code coverage and path depth detection ability. CVDF DYNAMIC generates its own test case sets through BI-LSTM neural network and genetic algorithm. Then, we integrate the two sample sets through the idea of ensemble learning to obtain a sample set with both code coverage and vulnerability mining ability for a critical execution path of the program. In order to improve the efficiency of fuzzy testing, we use heuristic genetic algorithm to simplify the integrated sample set. We also innovatively put forward the evaluation index of path depth detection ability (pdda), which can effectively measure the vulnerability mining ability of the generated test case set under the critical execution path of the program. Finally, we compare CVDF DYNAMIC with some existing fuzzy testing tools and scientific research results and further propose the future improvement ideas of CVDF DYNAMIC.

## 1. Introduction and Background

Vulnerability in program has always been a serious threat to software security, which may cause denial of service, information leakage and other exceptions. Some typical cases of vulnerability exploitation, such as wannacry ransomware, have a disastrous impact on social economy and network security. Therefore, mining vulnerabilities scientifically and efficiently has been a hot topic.

At present, vulnerability mining technology can be divided into static vulnerability mining and dynamic testing (fuzzy testing) [1]. The former does not construct test cases nor run source code. By extracting the characteristics or key operations of the corresponding types of vulnerabilities, static code audit is carried out on the source code to detect the possibility of various vulnerabilities. The target source code of static vulnerability mining can be advanced language, assembly language generated by compiler, or binary file. The advantages of static vulnerability mining lie in fast mining speed, high efficiency, and good detection accuracy for vulnerabilities with obvious characteristics. However, static vulnerability mining often leads to high false positive rate and false negative rate for vulnerabilities with unclear features or diverse types and forms (such as null pointer reference vulnerability in C/C++). Dynamic fuzzy testing can solve this problem by constructing reasonable test examples. However, the efficiency of dynamic fuzzy testing is lower than that of static vulnerability mining because it needs to construct samples and run programs to determine whether there are vulnerabilities. Therefore, how to construct test cases with high pdda and code coverage is the key of fuzzy testing. In practical application, it is often necessary to combine static vulnerability mining with fuzzy testing to achieve better vulnerability detection performance. Existing mainstream fuzzy testing can be divided into the following three categories:

- Black box test (construct test cases to test without source code at all);
- White box test (analyze source code to generate corresponding test cases); e.g., [2];
- Grey box test (introduce lightweight program analysis technology to analyze program state), e.g., [3].

In black box test, the internal structure of the program is not understood at all, and the test cases are constructed blindly. Thus, its testing efficiency is very low. White box test uses program analysis methods [4] (such as path traversal and symbolic execution) to analyze the program source code and then constructs the corresponding test cases. The white box test can cover deeper test path, which causes a lot time cost and system resources with poor scalability. The grey box test [5] can achieve a good balance between the test efficiency and the coverage of test cases because of the introduction of lightweight program analysis technology. It is more effective than a black box test and more extensible than a white box test. At present, the grey box testing program is mainly guided by code coverage. The typical grey box fuzzers are AFL [6] and so on. 

However, the problem of current grey box fuzzers is that they are designed to cover as many code execution paths as possible. In the regulation of seed energy, they usually use the idea of average distribution instead of regulating different energies for different test paths. Nevertheless, most of the source code vulnerabilities are concentrated on a small number of critical test paths in reality. Existing grey box fuzzers often spend a lot of time to detect the path whose vulnerability is not easy to be detected, thus reducing the efficiency of fuzzy testing.

Because the application of a single method in grey box fuzzy testing has its own limitations, more and more researchers have begun to integrate a variety of methods to achieve better fuzzy testing results, such as [7].

Based on existing research work [8], this paper proposes a new framework of fuzzy testing sample generation called CVDF DYNAMIC. It consists of three parts:

(1) The strategy of sample generation based on a genetic algorithm;
(2) The strategy of sample generation based on a bi-LSTM neural network;
(3) The strategy of sample reduction based on a heuristic genetic algorithm.

The genetic algorithm can improve the quality of test cases and expand the code coverage by simulating the natural process of gene recombination and evolution. The bi-LSTM time sequence can regulate different energy of the test path, which can make the seeds on the critical path iterate and mutate for many times, and enhance the path depth detection ability. The critical contribution of CVDF DYNAMIC is that it integrates the two methods of sample generation, and simplifies the sample set by using a heuristic genetic algorithm, which makes the test case set achieve a good balance in code coverage, path depth detection ability and sample set size. This paper also compares the proposed method with other fuzzy testing samples and further presents the improvement direction of that method.

## 2. Related Work

At present, researchers have applied fuzzy testing to different types of vulnerability mining. Lin et al. [9] proposed a priority-based path searching method (PBPS) to utilize the capability of concolic execution better. Peng et al. [10] proposed Angora, a new mutation-based fuzzer, and proved that Angora has better performance than other fuzzing tools. Wang et al. [8] used a neural network to guide the sample generation of fuzzy testing and proposed a solution called NeuFuzz. NeuFuzz has a very significant performance in the vulnerability mining of the critical execution path of the program. Zhang et al. [11] summarized existing fuzzy testing technologies and use case generation technologies of fuzzy testing. Zhang el al. [12] proposed an algorithm of sensitive region prediction based on a neural network and improved the detection efficiency and detection depth through the incremental learning method of sensitive areas. Combining fuzzy testing and symbolic execution, Xie [13] proposed a hybrid testing method based on a branch coverage called AFLeer. Xu et al. [14] applied a recurrent neural network to fuzzy testing sample generation. Luca et al. [2] designed a novel concolic executor to improve the efficiency of concolic execution and investigate whether techniques borrowed from the fuzzing domain can be used to solve the symbolic query problem. Stefan [15] proposed the notion of coverage-guided tracing to improve the efficiency of code coverage guided fuzzy testing. Yang et al. [16] proposed a novel programmable fuzzy testing framework. Developers only need to write a small number of fuzzy testing guidance programs to implement customized fuzzy testing. Patrice et al. [17] proposed learn&fuzz, which used a learned input probability distribution to intelligently guide fuzzing inputs. Li et al. [18] proposed symfuzz, which is a method combining directed fuzzy testing technology with selective symbolic execution technology and can realize vulnerability detection under complex path conditions. Liang et al. [19] proposed a machine-learning-based framework to improve the quality of seed inputs for fuzzing programs. Zou et al. [1] described the development from traditional automation to intelligent vulnerability mining in software vulnerability mining. This paper also pointed out that the application of traditional machine learning technology in the vulnerability mining field still has limitations. Ma et al. [20] proposed the optimization strategy of sample set reduction in the fuzzy process, including approximation algorithm. Cornelius [21] proposed IJON, an annotation mechanism that a human analyst can used to guide the fuzzer.

In the experimental part, this paper compares the simplification and efficiency of sample set between heuristic genetic algorithm and approximation algorithm. He et al. [22] proposed a tool called VCCFinder to find potential vulnerabilities. Nick et al. [23] used mined vulnerabilities by utilizing a code attribute graph for fuzzy testing. She et al. [24] proposed a novel program smoothing technique using a surrogate neural network models to achieve higher edge coverage and improve the ability of finding new bugs. Chen et al. [25] proposed POLYGLOT, a genetic fuzzing framework that generates high-quality test cases for exploring processors of different programing languages. Huang et al. [4] proposed PANGOLIN, an approach based on polyhedral path abstraction, which preserves the exploration state in the concolic execution stage and allows more effective mutation and constraint solving over existing techniques. Zhang et al. [26] proposed a novel incremental and stochastic rewriting technique STOCHFUZZ that piggy-backs on the fuzzing procedure. Liang et al. [3] presented DeepFuzzer, an enhanced greybox fuzzer with qualified seed generation, balanced seed selection and hybrid seed mutation. Chen et al. [7] proposed an ensemble fuzzing method, EnFuzz. Enfuzz contains many different heuristic genetic algorithms and achieves a better performance in terms of path coverage, branch coverage and bug discovery. The idea of ensemble is also similar to the CVDF DYNAMIC proposed in this paper. Yue et al. [27] presented a variant of the adversarial multi-armed bandit model for modeling AFL’s power schedule process named EcoFuzz, which can effectively regulate seed energy in fuzzy testing. Zong et al. [28] proposed FuzzGuard, a deep-learning-based approach to predict the reachability of inputs and further improve the performance of DGF. Gan [5] proposed a data flow sensitive fuzzing solution GREYONE, which can further improve the performance of data flow analysis, and the experiments show that GREYONE has better performance than the existing fuzzy testing tools such as AFL. Sebastian et al. [29] proposed ParmeSan, a sanitizer guided fuzzing method to solve the low-bug coverage problem. Oleksii et al. [30] proposed specfuzz, which is a novel fuzzy testing method, which can be used to detect speculative execution vulnerabilities including spectre and out-of-order execution vulnerabilities. Compared with the traditional static analysis method, specfuzz has further improved the analysis accuracy. Lee et al. [31] proposed a constraint-guided directed greybox fuzzing method, which aims to satisfy a sequence of constraints rather than merely reaching a set of target sites. Christopher et al. [32] proposed a brand-new token-level fuzzing method. Different from the fuzzy method based on data flow or seed energy regulation, token-level fuzzing applies mutations at the token level instead of applying mutations either at the byte level or at the grammar level. The authors found many unknown bugs through the token-level fuzzing method on popular javascript engines. In recent years, the safety of deep learning technology has also attracted the attention of scholars. It is possible for attackers to deduce the sensitive training data of engineering through the unsafe deep learning model. Ximeng Liu et al. [33] briefly introduced four different types of attacks in deep learning, reviewed and summarized the security defense measures of deep learning attack methods and further discussed the remaining challenges and privacy issues of deep learning security. MB mollah et al. [34] proposed an efficient data-sharing scheme, which allows smart devices to share secure data with others at the edge of cloud-assisted IOT.

## 3. Algorithm Description

### 3.1. An Introduction of Existing Fuzzy Testing Sample Generation Methods

At present, the generation and variation methods of test cases are mainly described as follows:

The method based on symbolic execution [13].

The core idea of this method is to take the test case as the symbol value and search the core constraint information on the test path during the processing. A new test case is generated by constraint solving to cover different program execution paths. This method is suitable for testing programs with simple structure and less execution paths. However, the complexity of the program increases with the diversification of functions, resulting in the explosion of the number of paths. It is difficult for symbolic execution to be applied to constructing complex program test cases because of complex constraint solving problems.

The method based on taint analysis [10].

The core idea of this method is to mark the pollution source of the input data by using the dynamic taint analysis technology, focus on the spread process of the taint, extract the key taint information from it and use the taint information to guide the generation of seed variation and related test samples. It is an effective method to construct test samples for some key execution paths in programs and has good code coverage, such as Angora [10]. However, with the application of genetic algorithm and neural network in fuzzy testing, the disadvantage of low efficiency of taint analysis technology is gradually emerging.

The method based on evolutionary algorithm [35].

The evolutionary algorithm uses some core rules of biological evolution to guide the generation of fuzzy testing samples. At present, genetic algorithm is the most widely used evolutionary algorithm with the best performance. Its core idea is to carry out multiple rounds of iterative mutation on test cases, eliminate the test cases that do not meet the requirements according to some rules or select the samples with the best performance from them as the seeds of the next round of mutation. Genetic algorithm can be used not only to generate new test cases but also to simplify the sample set, so as to further improve the efficiency of fuzzy testing.

The method based on neural network [14].

As mentioned above, neural network has a very significant performance advantage in solving some nonlinear problems. The bi-LSTM neural network is used to mutate the seeds on a certain execution path to obtain a new test example. In the experiment, we prove that the bi-LSTM neural network has stronger path depth detection ability in specific key execution paths than that of the taint analysis. Moreover, Learn & Fuzz proposed by Patrice [17] et al. can improve the code coverage of fuzzy testing. Therefore, it can be predicted that the neural network will play a greater role in the future development of fuzzy testing.

### 3.2. Formal Definition

In order to facilitate the subsequent description of the algorithm, we give some related concepts and formal definitions of the evaluation index.

- Definition 1 PUT (input sample)

We define the program under test as PUT. For CVDF DYNAMIC, PUT is the corresponding binary executable program, and the corresponding test cases are mentioned in Section 4.1.

- Definition 2 Set Covering Problem (SCP)

A large number of facts show that there is an exponential proportional relationship between the growth number of execution paths of PUT and the growth number of its branch conditions, so the test cases cannot completely cover all execution paths. Therefore, in fuzzy testing, the problem of sample set coverage is transformed into the problem of minimum set coverage [36]. The minimum set covering problem is an NP hard problem [37]. The simplest algorithm idea is to use greedy algorithm to find the approximate optimal solution. The following formal definition is used to describe SCP problem:

For $A=[a_{ij}]$, it is a 0–1 matrix of m-row n-columns, where $C=C_{j}$ is an n-dimensional column vector. Let p=[1,2,3……m] and q=[1,2,3……n] be the row and column vectors of matrix A. Furthermore, let $C_{j},j\in q$ represent the cost of a column. Without losing generality, we assume that $C_{j}>0,j\in q$. It is specified here that if $a_{ij}$ = 1, it means that column j∈q at least covers one row i∈p. Therefore, the essence of the SCP problem is to find a minimum cost subset S⊆q. So, for every row i⊆p, it is covered by at least one column j⊆S. A natural mathematical model of SCP can be described as $v(SCP)=\min\sum_{j\in q}C_{j}x_{j}$, and it obeys $\sum_{j\in q}a_{ij}x_{j}\geq 1,i\in p,x_{j}\in (0,1)(j\in q)$. If $x_{j}=1(j\in S)$, then $x_{j}=0$.

- Definition 3 Path Depth Detection Ability

In fuzzy testing, there are many program-execution paths that may have vulnerabilities in PUT, so the generation of fuzzy testing samples should cover as many as possible for these program execution paths that may have vulnerabilities. For a program execution path, the number of detected vulnerabilities may be more than one, and different program execution paths can detect different numbers of vulnerabilities. We define the total number of vulnerabilities detected by the fuzzy testing sample under the current path as $D_{NUM}$, the total number of vulnerabilities contained in the current path as $A_{NUM}$ and the weight of the total number of vulnerabilities contained in the current path as W. DetectionCapability(DC) is a weighted result, and its operation method is shown in Equation (1):

(1) $$DC=\frac{D_{NUM}}{A_{NUM}}\times W$$

Among them, W increases with the number of vulnerabilities in the current path. This is because the number of vulnerabilities in different paths is different. For the variation method of the same fuzzy testing sample seed, if more vulnerabilities are contained in a path, the smaller the value of $\frac{D_{NUM}}{A_{NUM}}$ is. If the weight W is a constant, the DC value will decrease, and the path depth detection ability of a test case generation method cannot be objectively measured.

Suppose that a program under test has n execution paths, we define the average path detection ability as $WDC=\frac{\sum_{i=1}^{n}DC_{i}}{n}$: It can measure the ability of a fuzzy testing tool to detect the overall path depth

### 3.3. CVDF DYNAMIC Fuzzy Testing Sample Generation

**Figure 1:**
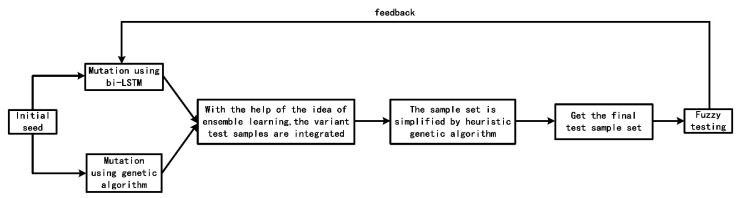
Complete Flow Chart of CVDF DYNAMIC Fuzzy Testing sample generation.

The complete process of fuzzy testing sample generation of CVDF DYNAMIC is shown in Figure 1.

In the fuzzy testing part, we learn from the ensemble learning method in artificial intelligence. The seeds are mutated by genetic algorithm to generate a set of test cases, and then the seeds are mutated by the bi-LSTM neural network to generate another set of test cases. Finally, the two sets of test cases are integrated to obtain the final set of test cases.

Considering that the size of the sample set obtained by the integration of the two methods is too large, which reduces the efficiency of fuzzy testing, we use heuristic genetic algorithm to simplify the sample set. Finally, the reduced sample set is used for fuzzy testing, and the parameters in the bi-LSTM neural network are optimized according to the result feedback.

#### 3.3.1. Theoretical Model and Training Process of BI-LSTM Neural Network

**Figure 2:**
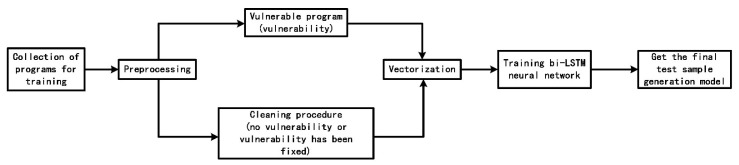
Training of neural network.

The BI-LSTM neural network training process of CVDF DYNAMIC is shown in the Figure 2.

(a) Preprocessing and Vectorization

We preprocess the training dataset, including unifying the input format of the test cases and changing the format of some binary executable programs, so that they can adapt to the input of the neural network without changing the logic function of the original program.

Then, we use the PTFuzz tool, which is a tool to obtain the program execution path by using the Intel Processor Tracing module (IntelPT). PTFuzz makes a further improvement on the basis of AFL, which removes the dependence on the program instrument but uses PT to collect package information and filter package information, and finally obtains the execution path of the current seed according to the package information. In order to achieve this goal, our hardware environment should be based on Intel CPU platform and run under the appropriate version of Linux system. Since the PTFuzz tool stores the program execution path information in data packets in order to obtain the program execution path information that can be trained for neural networks, we need to decode the data packets in the corresponding memory and recover the complete program execution path according to the entry, exit and other relevant information of each data packet. The pseudocode of the Algorithm 1 Extracting program execution path is as follows:
**Algorithm 1. Extracting program execution path****Start Func** **Func ExtractPath**(binary-source-code) 1: Start = LoadBinaryProgram(binary-source-code) 2: ProgStaddr = GetProgramEntry(Start) 3: ExecutionPath = [] 4: **while** True: 5:   PackagePath = LoadCurrentPackage(ProgStaddr) 6:    ExecutionPath +|= PackagePath 7:   **If** ProgStaddr == JumpNextInstrument() 8:      ProgStaddr = GetNextInstruAddr() 9:   **If** ProgStaddr == EndOfMemSpace() 10:    **break** 11: **Return** ExecutionPath **End Func**

In the pseudocode, JumpNextInstrument() and EndOfMemspace() are two judgment functions, which are used to judge whether to jump to the next instruction address and whether the end of the memory address of PTFuzz package has been reached, respectively. The ExecutionPath variable forms a complete program execution path by continuously connecting the PackagePath variable after decodeding. +|= is a concatenate operation.

After extracting the program execution path, we need to convert the program execution path containing instruction bytecodes into vector form and save the original semantic information of the original program execution path as much as possible.

**Figure 3:**
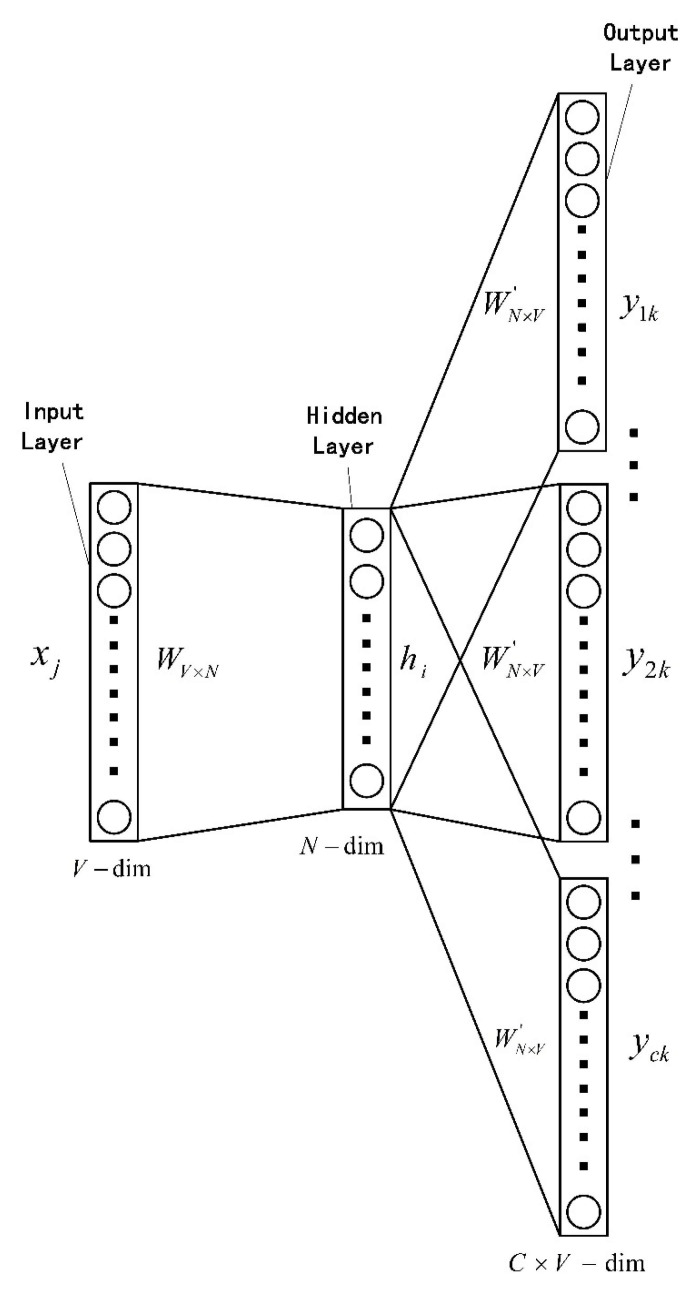
The Basic Structure Diagram Of Skip-Gram Model.

We use the tool word2vec and regard a complete program execution path as a statement and an instruction as a word. Specifically, we regard the hexadecimal code of an instruction as a token, and then we use word2vec to train the corresponding bytecode sequence. In order to preserve as much context information as possible in the program execution path, we choose the Skip-Gram model in word2vec because it often has better performance in large corpus. The Skip-Gram model structure is shown in the Figure 3.

Finally, we need to transform the output of word2vec into an equal length coding input, which can be used as the input vector of the neural network. Let us set a maximum length, which is MaxLen. When the output length of word2vec is less than MaxLen, we use 0 to fill in the back end to make it MaxLen. When the output length of word2vec is larger than MaxLen, we truncate it from the front end and control the length to MaxLen.

(b) BI-LSTM neural network structure and parameter optimization

The neural network structure we choose is bi-LSTM.

**Figure 4:**
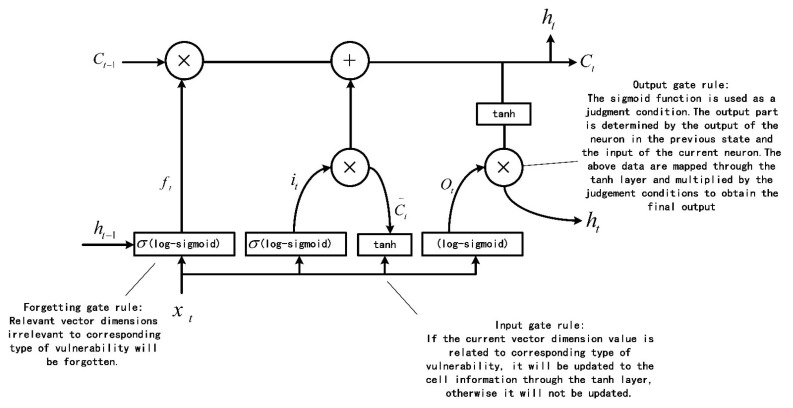
The Specific Structure of LSTM Neuron.

Bi-LSTM has excellent performance in dealing with long-term dependency problems, such as statement prediction and named entity recognition [38]. The statements associated with vulnerability characteristics may be far away in the whole program execution path, so we need the bi-LSTM neural network structure for the long-term memory of the information related to the vulnerability characteristics. In order to make the bi-LSTM neural network suitable for fuzzy testing, we modify the corresponding rules of the input gate, output gate and forgetting gate of the bi-LSTM. The specific structure of the single LSTM neuron and the specific rules of the input gate, output gate and forgetting gate are shown in Figure 4.

**Figure 5:**
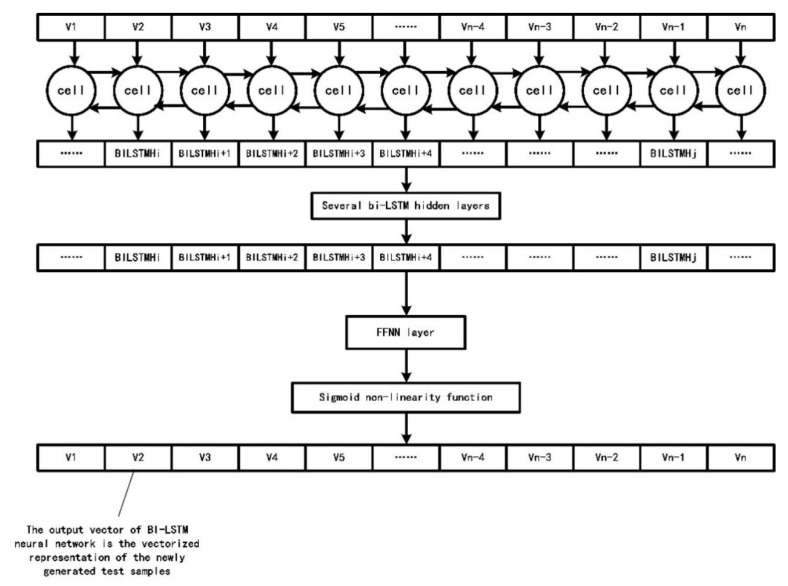
The Complete Structure of the bi-LSTM neural network.

The number of hidden layers in the bi-LSTM neural network, epochs, batch size and other parameters will affect the final performance of the neural network. According to the experimental part in Section 4.2, we set the number of hidden layers to 5, the batch size to 64 and the drop rate to 0.4 and use a BPTT back-propagation algorithm to adjust the network weight, using random gradient descent (SGD) method to prevent the model from falling into the local optimal solution. For the hyper parameters in the bi-LSTM neural network, we choose to use dichotomy to accelerate the selection of corresponding values. Figure 5 shows the complete structure of the bi-LSTM neural network.

From Figure 5, we make the coding input with length MaxLen pass through several bi-LSTM hidden layers to extract clearer context dependencies. We let the output of the last bi-LSTM hidden layer pass through a feed forward neural network layer and sigmoid activation function. The sigmoid activation function also normalizes the final output vector, which is the vector form of the fuzzy testing sample generated by the bi-LSTM neural network.

#### 3.3.2. Genetic Algorithm for Constructing Test Cases

**Figure 6:**
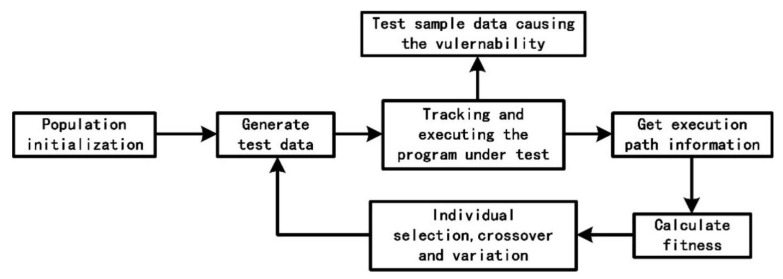
General Flow Chart of Generating Test Cases By Genetic Algorithm.

The core of the genetic algorithm used to construct samples can be divided into several parts, including population initialization, tracking and executing the tested program, fitness calculation and individual selection, crossover and mutation. The overall structure is shown in Figure 6.

(a) Population initialization

In a genetic algorithm, the population is composed of several individuals. We abstract an individual as a chromosome. Let us set the length of the chromosome as $D_{len}$, which means the number of bytes of test data. Then, the ith individual in the population can be expressed as $X_{i}=(x_{i,1},x_{i,2},x_{i,3},\ldots ,x_{i,D_{len}})$. Population initialization is performed to assign a value to each gene $x_{i,k}$$\left(1\leq k\leq D_{len}\right)$ in $X_{i}$. When there are initial test data, each byte of the initial test data is used to assign a value of $x_{i,k}$. Otherwise, the whole population can be initialized by randomized assignment.

(b) Tracking and executing the program under test

Tracking is divided into two aspects:

- Monitor whether the current test data will cause the tested program to crash;
- Record the execution path of the program

Because each program can be divided into many basic blocks during execution, the essence of the program execution is the process of execution and jump between basic blocks.

Each basic block has only one entry and exit. So, in a basic block, the program enters from the entry and exits from the exit. Therefore, we can use the entry address Inaddr of the basic block to represent each basic block. Then, the program execution process can be expressed as a sequence of basic blocks: $\left(Inaddr_{1},Inaddr_{2},\ldots ,Inaddr_{n}\right)$ We define the jump of a basic block as $e=(Inaddr_{k},Inaddr_{k+1})$, where (1≤k≤n−1).

Obviously, if every basic block is regarded as a point in a graph, then E is an edge in the graph. Since a basic block may be executed multiple times in the execution sequence, the graph is directed. In this case, the execution path of the program can be expressed as a sequence of edges $E_{e}=(e_{1},e_{2},e_{3}\ldots e_{n-1})$.

Because some basic blocks may be repeated many times during program execution, some edges may appear many times. We combine the same edges to obtain a set of edges with the information of times of occurrence and analyze the frequency statistics of this set and further divide it into many groups according to the different times of occurrence 1, 2–3, 4–7, 8–15, 16–31, 32–63, 64–127 and 128.

It is easy to see that the significance of this classification is that it can use different bits of a byte to represent the times information, so it can improve the processing speed of the program. Finally, we will obtain a new set of occurrence information $F_{e}=(f_{1},f_{2},f_{3}\ldots f_{n-1})$.

We use the above processing method for each basic block to get the final program execution path information.

(c) Fitness calculation

By tracking the program under test, we can see that an execution path information can be expressed as a sequence of edges. Therefore, in order to find a new execution path and improve the path coverage of CVDF DYNAMIC, we need to calculate the fitness. We define the sequence set of edges as $V=(V_{1},V_{2},\ldots ,V_{n})$, where each $V_{k}$ (1≤k≤n) is equivalent to $E_{e}$. For any edge in $E_{e}$, let us assume that the final test data are $X_{i}$. We can obtain a binary set of edge information related to the test data, as shown in Equation (2):

(2) $$Q_{i}=\{(e_{i,1},X_{i,1}),(e_{i,2},X_{i,2})\ldots (e_{i,n},X_{i,n})\}$$

It is not difficult to find that its essence is a weighted digraph, and the weight is the test data. We define that the fitness (adaptation) f of an individual consists of two functions, as shown in Equations (3) and (4).

Finding the number of new edges $f_{1}$ and the number of edges $f_{2}$ associated with them in $Q_{i}$:

(3) $$f_{1}(X_{i})=card(V_{i}-E_{t})$$

(4) $$f_{2}(X_{i})=\sum_{q\in V_{i}}^{q}G(W_{q},X_{i})$$

(5) $$G\left(X_{1},X_{2}\right)=\{\begin{array}{l} 1\left(X1=X2\right)\\ 0\left(X1\neq X2\right) \end{array}$$

Firstly, the fitness $f_{1}$ of each individual is calculated, and then the fitness $f_{2}$ of each individual is calculated after updating the set. The two sets used to calculate the fitness are updated after each round of testing. When comparing two individuals, first $f_{1}$ is compared; if $f_{1}$ cannot be distinguished, then compare $f_{2}$.

(d) Individual selection, crossover and variation

Our individual selection method uses elite selection to produce new individuals. It is a strategy of generating new individuals in genetic algorithm, which makes the individuals with high fitness enter the next generation. The method of crossover is 2-opt transformation. A number of random numbers are generated as the intersection points, and then the fragments of the intersection points in the chromosome are exchanged. Rather than using the random mutating method, this paper proposes a control mutation method to improve the effect of mutation. A motivating example of the Algorithm 2 Control Mutation is as follows:
**Algorithm 2. Control Mutation****Start Func** **Func ControlPROC**(X,Y) 1: A = 1, B = 1 2: **IF** Y >= B **THEN** 3: **FORK1:** A = A × X, B = B + 1 4: **ELSE:** 5:   **IF** X >= A **THEN** 6:      FORK2: A = A + X, B = B − 1 7:   **ELSE:** 8:      FORK3: A = A − X, B = B/2 9: **RETURN** A **End Func**

The input data format of the program is (X,Y) assuming that the template data are (X=1,Y=1), and the variation factor is the operation of replacing 0. Therefore, two test data can be generated by mutation (X=1,Y=0) and (X=0,Y=1), which can cover FORK1 and FORK2. This form of testing could not achieve 100% branch coverage due to the failure to cover FORK3. For control variation, when the test data (X=1,Y=0) generated by the variation make the program enter the new branch FORK2, the variation field of this time will be marked as an immutable field, and the variation will be carried out on the basis of the test data. In this example, the control variation marks Y=0 as an immutable field and mutates the remaining fields, the X value, to 0, resulting in test data (X=0,Y=0) that can be overridden by FORK3.

The control mutation strategy consists of the test data and control information that make the program enter the new branch. The control mutation process is as follows: Firstly, the control mutation strategy is taken out from the policy database, and the test data entering the new branch are taken as the mutation template. Secondly, check the stored control information and each byte in the template to confirm whether it is marked as control information; if so, check the next byte, if not, modify the byte in combination with random mutation strategy, generate test data and execute fuzzy testing, then continue to check the next byte. Finally, after all bytes are checked, we complete one time of mutation, and the above process is repeated.

After completing the above operations, we have completed a round of iteration of the genetic algorithm taking the newly generated chromosome data as the test data of the next round of mutation, that is, continuous iterative mutation.

#### 3.3.3. Integrating New Test Data with Integration Idea

Firstly, through the above genetic algorithm, test cases with high path coverage are constructed from the original test case seeds. Then, for the test cases located on different execution paths, the bi-LSTM neural network is used to construct test cases with stronger path depth detection ability. Finally, we integrate the test case set constructed by the two methods to obtain the final test case set. Considering that the test case set generated by the above two methods may be too large and the efficiency of the fuzzy testing is reduced, this paper uses heuristic genetic algorithm to simplify the integrated test case set to ensure that the efficiency of fuzzy testing can be improved without losing the test performance.

#### 3.3.4. Using Heuristic Genetic Algorithm to Reduce Sample Set

In order to reduce the sample set without losing the performance of fuzzy testing as much as possible, the screening principle of heuristic genetic algorithm in this paper is to give priority to the samples with stronger code coverage and Path Depth Detection Ability. Then, select the remaining test samples in the order of decreasing test performance, until the performance index basically covers the original fuzzy testing sample set (see the experiment in Section 4.4 for specific results). Here, our heuristic algorithm is a selection mutation algorithm for chromosomes.

(a) Using a compression matrix to represent chromosomes

At present, the common chromosome representation method is to use a 0–1 matrix [39]. The element of each row vector of the 0–1 matrix is 0 or 1. As mentioned earlier, we treat the basic block address as a collection of elements. Each basic block is equivalent to the gene in the genetic algorithm. Therefore, 1 in the 0–1 matrix indicates that a basic block exists in the sample, while 0 indicates that it does not exist. In this way, the sample set formed by all samples constitutes a 0–1 matrix, and the set of genes in each column is equivalent to a chromosome. Considering the complexity of the program execution path, the 0–1 matrix is a sparse matrix. If it is stored directly in the way of 0–1, the space efficiency will be significantly reduced. Therefore, this paper compresses the 0–1 matrix. Our storage method is a triple sequence $<Val,X_{cor},Y_{cor}>$, where Val is the element with the storage value of 1, and $X_{cor}$ and $Y_{cor}$ are its X and Y coordinates in the original matrix, respectively. Since the value of Val is 1 by default, the value of this item can be omitted in the actual operation.

(b) Using heuristic genetic algorithm to improve chromosome

Each chromosome has its own independent gene sequence, but there will also be a large number of repeated and overlapping genes. Therefore, as mentioned above, we should solve the SCP when carrying out set coverage and reduce set redundancy as much as possible. Therefore, the heuristic function of the heuristic genetic algorithm is mainly reflected in eliminating the redundancy caused by gene duplication and screening better chromosomes through genetic iteration.

The specific algorithm is described as follows:

We deduce the chromosome from the position information in the compression matrix. For genes in the same column, if they contain more “1” values, it indicates that the performance priority of this column is relatively high, so we give priority to selection, mark the selected column and so on. Subsequently, we perform gene exchange on chromosomes. We assume that there are two different chromosomes $Fa_{1}$ and $Fa_{2}$ in the parent generation. After chromosome exchange, we can obtain the child’s chromosomes $Ch_{1}$ and $Ch_{2}$. It is assumed that $Ch_{1}$ and $Ch_{2}$ can cover set $S_{1}$. We use sets $T_{1}$ and $T_{2}$ to store the line numbers not covered in the genes and use sets $Cot_{1}$ and $Cot_{2}$ to store the genes contained in $Ch_{1}$ and $Ch_{2}$. First, we calculate the performance priority of each gene in the parents $Fa_{1}$ and $Fa_{2}$, that is, count the number of “1” values in each column for screening. Then, we screen out the chromosomes with the highest performance priority in $Fa_{1}$ and $Fa_{2}$, copy them to $Ch_{1}$, count the genes contained in $Ch_{1}$ and delete the genes contained in $Ch_{1}$ from $Cot_{1}$. Then, we calculate the value of $Cot_{1}-Ch_{1}$, which is the difference set, and store its line number in set $T_{1}$. Next, we continue to arrange the remaining genes of $Fa_{1}$ and $Fa_{2}$ using the same performance priority selection method, and then put them into $Ch_{1}$ again. The remaining genes will be put into $Ch_{2}$.

In the process of gene selection and gene exchange, there are some special cases with the same gene performance. At this time, we need to further screen them to obtain the optimal gene. Suppose that there are two genes, $Gene_{1}$ and $Gene_{2}$, with the same performance priority in $Fa_{1}$, and there is one gene $Gene_{3}$ in set $Ch_{1}$. At this time, we need to compare the results of $Gene_{1}\cap Gene_{3}$ and $Gene_{2}\cap Gene_{3}$ to screen out the larger results. Considering that there will be a corresponding mutation process in the genetic algorithm, the above calculation should be carried out before and after mutation to ensure that the optimal result is always selected.

From the above description, the heuristic genetic algorithm proposed in this paper uses the compression matrix on the basis of the original population and selects the optimal chromosome according to the way of gene selection and gene exchange. Therefore, this heuristic genetic algorithm essentially does not change the workflow of ordinary genetic algorithm, but through the optimization of search conditions, it simplifies the sample set and further improves the efficiency of fuzzy testing.

The specific process of the ordinary genetic algorithm has been described above. The heuristic genetic algorithm is different from ordinary genetic algorithm in the following aspects:

(c) Paternal selection

There are three common methods of paternal selection: random selection, tournament selection and roulette bet. Here, we use roulette method, the specific operation is as follows:

Step 1: The fitness of each individual in the population is calculated *fi* (*i* = 1,2,3,…*n*), where n is the population size.

Step 2: Calculate the probability $p_{i}=\frac{f_{i}}{\sum_{1}^{n}f_{i}}$ of each individual being inherited into the next generation population.

Step 3: Calculate the probability distribution of each individual:

(6) $$q_{i}=\sum_{j=1}^{i}p(x_{j}).$$

Step 4: A pseudo-random number (rand) with uniform distribution is generated in the interval (0,1).

Step 5: When $rand<q_{1}$, $q_{1}$ is chosen; otherwise, if $q_{k-1}\leq rand\leq q_{k}$, individual K is chosen.

Step 6: Repeat step 4 and step 5 several times, and the number of repetitions depends on the size of the population.

(d) Cross rate selection

Crossover is the main way to produce new individuals. The crossover rate is the number of chromosomes in the crossover pool. A reasonable crossover rate can ensure that new individuals will be produced continuously in the crossover pool, but it will not produce too many new individuals, so as to prevent the genetic order from being destroyed. This paper adopts the most popular method of the adaptive crossover rate.

(e) Variation rate selection

The mutation rate is the proportion of the number of genes in a population based on the number of all genes. Because mutation is a way to produce new individuals, we can control the mutation by setting the number of genes or the rate of random mutation. Too low a mutation rate will lead to too few chromosomes involved in the mutation, which leads to the problem that the chromosome containing unique genes cannot be entered into the set. The high mutation rate will cause too many chromosomes involved in the mutation, which will generate some illegal data and increase the time cost. After the experiment and model tuning, the final mutation rate is 0.5.

(f) Elite ratio

The elite ratio means that the individuals with the highest fitness in the current population do not participate in crossover and mutation operations but replace the individuals with the lowest fitness in the current population after crossover and mutation operations.

After the experiment and model optimization, the final elite ratio is 0.06.

(g) Stopping Criteria

The genetic algorithm has to go through several rounds of iterative evolution until it reaches the ideal result or reaches the threshold of the number of iterations. For the heuristic genetic algorithm, the threshold of iterations is 25.

## 4. Experiment and Evaluation

### 4.1. Data Sources

In the training part of the neural network, we need a large number of training samples to train our neural network so that the time series neural network can effectively capture the corresponding kinds of vulnerability characteristics from the training set. Therefore, we first collect a large number of vulnerability information from CVE and CNNVD national security vulnerability database, then screen out the vulnerability information, which is obviously suitable for neural network training. Then, we select the corresponding binary executable program and corresponding test cases from GitHub [40] and SARD [41] dataset and obtain a small number of training datasets from Symantec Security Company. The dataset we screened contains a variety of CWE vulnerability types, such as buffer overflow vulnerability (CWE-119, CWE-120, CWE-131), format string (CWE-134), etc. For the binary executable program corresponding to each vulnerability information, we filter out two versions, which are vulnerable version (no patch version) and clean version (with patch version). The purpose of using two different versions to train the neural network is to verify whether the corresponding test cases can trigger the vulnerability successfully. Second, we can further enhance the learning of the neural network for vulnerability features through this method of comparative training, so as to achieve a better training effect. The inspiration for the construction of this training dataset comes from the special training dataset constructed for generator G in GAN neural network, which contains labeled samples and unlabeled samples. Finally, all the datasets we get are shown in Table 1.

We randomly select 80% of the data for the bi-LSTM neural network training set and the remaining 20% for CVDF DYNAMIC framework and subsequent experimental comparative analysis test set.

In the experiment, we mainly answer the following three questions:

Q1: Is the theoretical model of CVDF DYNAMIC valid?

Q2: Does CVDF DYNAMIC have a performance advantage in test case generation compared with the existing fuzzy testing tools?

Q3: What is the performance overhead of CVDF DYNAMIC? Does the reduction of sample sets improve the efficiency of CVDF DYNAMIC sample generation?

### 4.2. Evaluate the Validity of CVDF DYNAMIC’s Theoretical Model

**Figure 7:**
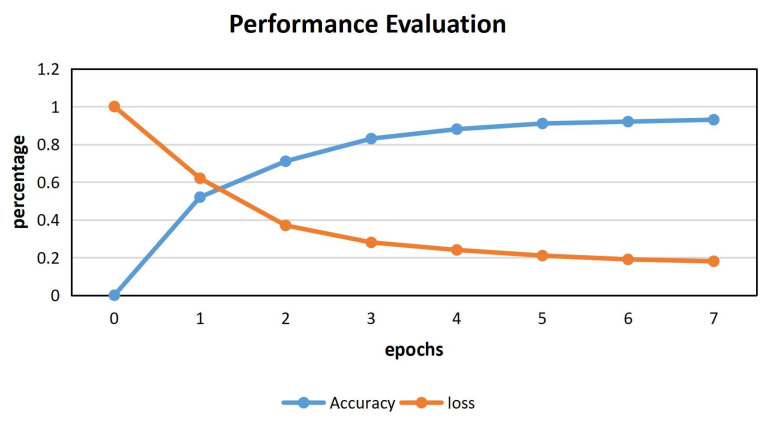
The Relationship Between the Accuracy and Loss Of bi-LSTM And Epochs.

For Q1, our BI-LSTM neural network optimizes the parameters according to the method mentioned above, and after seven epochs training, the accuracy and loss performance of the model are shown in Figure 7.

It can be seen from Figure 7 that after seven training epochs, the accuracy of the BI-LSTM neural network is more than 90%, approaching 93% and stable, while the loss is less than 20% and tends to be stable.

**Figure 8:**
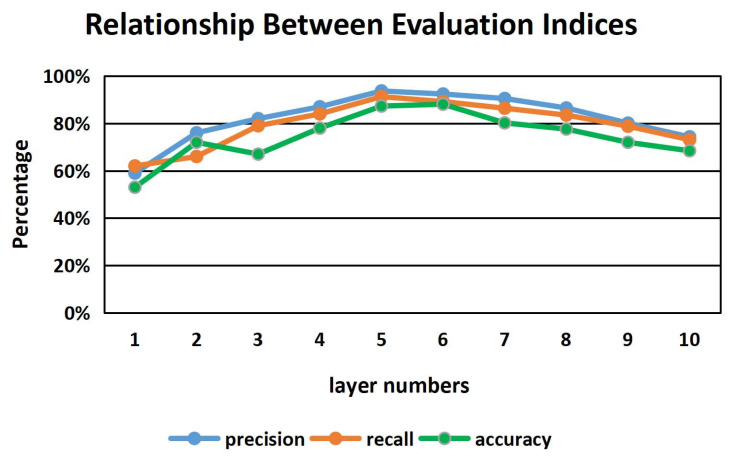
Relationship Between Evaluation Indices And Layer Numbers.

Figure 8 shows a specific example of parameter optimization for the number of hidden layers of the bi-LSTM neural network. As can be seen from Figure 8, when the number of hidden layers is five, the performance of the bi-LSTM neural network on the three evaluation indices of precision, recall and accuracy is the best. Other parameters such as drop rate and batch size are optimized in a similar way.

**Figure 9:**
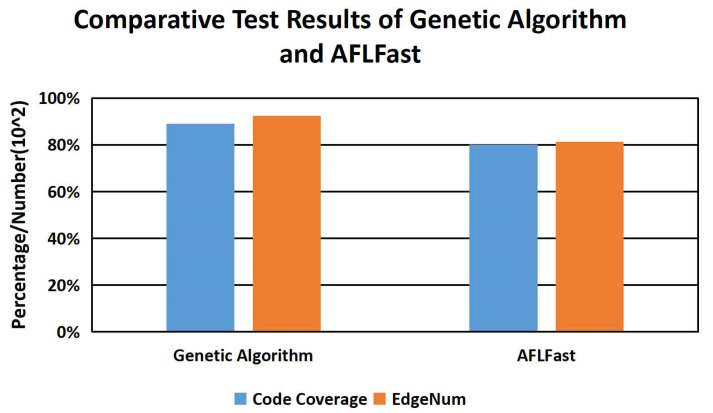
Comparative Test Results Of Genetic Algorithm And AFLFast.

In the part of using the genetic algorithm to generate test cases, we compare the genetic algorithm with the existing fuzzy testing tool AFLFast under the two evaluation indices of code coverage and the number of generated edge sequences EdgeNum. The genetic algorithm has been generated through 25 rounds of iterations, and the test program uses the media processing program named FFmpeg [42] in the test set constructed above. The final experimental results are shown in Figure 9.

In Figure 9, the ordinate dimension of code coverage is a percentage, and the dimension of the sequence number of edges is $value\times 10^{2}$. As can be seen from Figure 9, compared with AFLFast, the genetic algorithm has significant performance advantages in code coverage and the number of edge sequences. The genetic algorithm finds 9246 edge sequences for FFmpeg, while AFLFast only finds 8137 edge sequences. Because of the positive correlation between the number of edges and code coverage, the code coverage of the genetic algorithm is better than that of AFLFast.

So far, we have effectively solved the first problem, that is, the CVDF DYNAMIC theoretical model is effective. For the bi-LSTM neural network part of CVDF DYNAMIC, Figure 7 shows that our model achieves ideal training results. For the part of genetic algorithm generating test cases in CVDF DYNAMIC, our test cases have performance advantages over AFLFast in terms of code coverage and number of edges.

### 4.3. Performance Comparison between CVDF DYNAMIC and Existing Fuzzy Testing Tools

For Q2, we use NeuFuzz, which is also based on a neural network to guide the generation of fuzzy testing samples, and AFLFast tools for comparative testing. In order to facilitate testing and comparison, we use widely used evaluation metrics in vulnerability mining and neural networks, including false positive rate (FPR), true positive rate (TPR) and accuracy rate (ACC).

Firstly, the common definitions of vulnerability evaluation index are given.

TP (true positive): True positive samples are samples with their own vulnerabilities and are correctly identified.

FP (false positive): False positive samples are samples that do not contain vulnerabilities and are not correctly identified.

FN (false negative): False negative samples are samples that contain vulnerabilities and are not correctly identified.

TN (true negative): True negative samples are samples that do not contain vulnerabilities and are correctly identified.

The specific forms of FPR, TPR and ACC are as follows:

$$TPR=\frac{TP}{TP+FN}$$

$$FPR=\frac{FP}{FP+TN}$$

$$ACC=\frac{TP+TN}{TP+FP+TN+FN}$$

On the other hand, in order to intuitively show the performance advantages of the bi-LSTM neural network and genetic algorithm integration, we also add two evaluation indices, which are code coverage and path depth detection ability, and use the dataset constructed in this paper to test it. The experimental results are shown in Table 2.

It can be seen from Table 2 that CVDF DYNAMIC has performance advantages over other fuzzy testing tools. This is because CVDF DYNAMIC combines the advantages of neural network and genetic algorithm and is superior to other tools in comprehensive performance. However, other tools are also very advanced fuzzy testing tools, so they also have good performance in contrast testing. CVDF DYNAMIC and NeuFuzz are very close to each other in terms of other evaluation indices, except code coverage. However, CVDF DYNAMIC has obvious advantages over NeuFuzz in code coverage because it combines the advantages of the bi-LSTM neural network and genetic algorithm. It should also be pointed out here that the author of NeuFuzz explains that NeuFuzz focuses on seed mutation and test case generation under critical execution path rather than code coverage. However, CVDF DYNAMIC is still in the leading position in comprehensive performance.

### 4.4. Performance Overhead of CVDF DYNAMIC and Effectiveness of Sample Set Reduction

For Q3, we consider the performance cost of CVDF DYNAMIC and the effectiveness of sample set reduction from the number of sample sets before and after reduction, the time of fuzzy testing before and after reduction, the compression ratio and other evaluation indicators.

From Table 3, it can be seen that the compression algorithm greatly reduces the number of samples, and the compression rate reaches 54.6%. However, because the compressed sample set basically retains the key path, the execution time has decreased to some extent, but it is not as obvious as the compression rate. The code coverage and WDC evaluation index of the compressed sample set are identical with the original sample set. It shows that the compression of the test case sample set has no loss of performance, and then proves the significance and necessity of the sample set compression.

**Figure 10:**
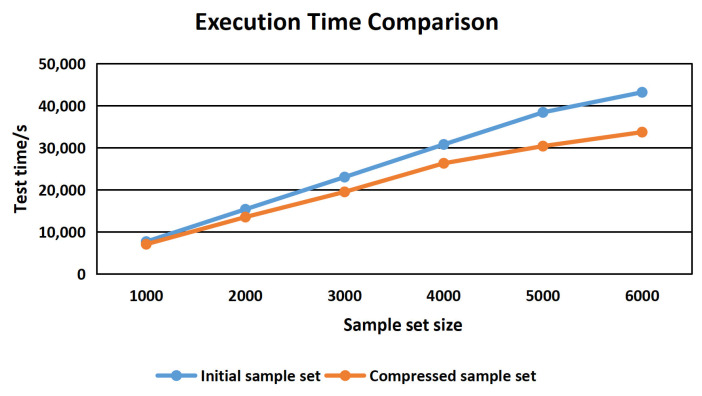
Execution Time Comparison.

We use a random sampling method to form 6 initial sample sets with the scales of 1000, 2000, 3000, 4000, 5000 and 6000. The execution efficiency and time of the initial sample set and of the compressed sample set are compared, and the results are shown in Figure 10.

As can be seen from Figure 10, with the increase in the initial sample set size, the execution time efficiency after compression is gradually improved compared with that before compression.

**Figure 11:**
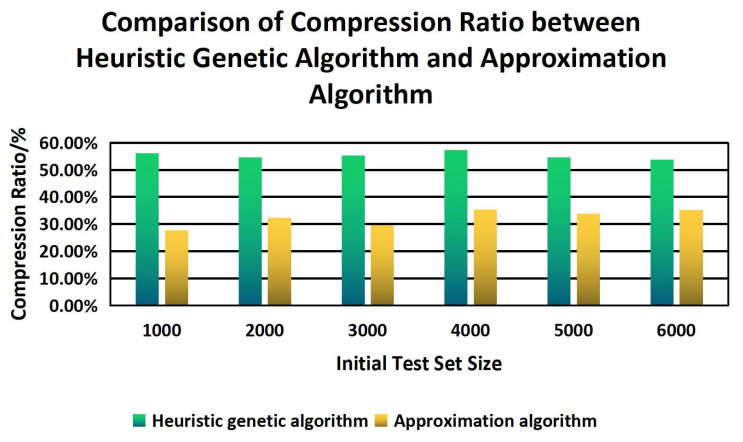
Comparison Of Compression Ratio Between Heuristic Genetic Algorithm And Approximation Algorithm.

**Figure 12:**
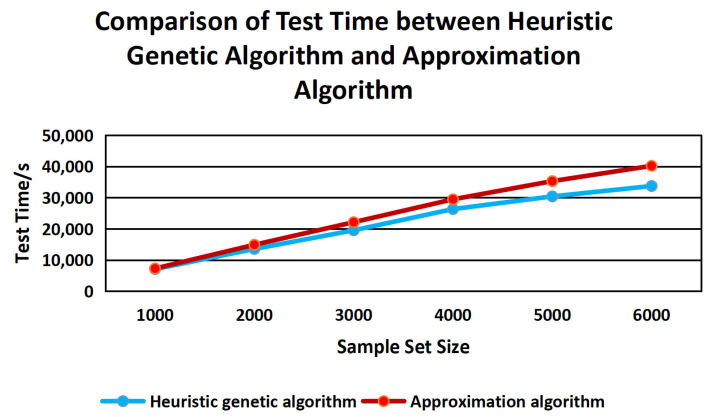
Comparison of Test Time Between Heuristic Genetic Algorithm And Approximation Algorithm.

Finally, this paper compares the compression ratio and test time of the sample set between the CVDF DYNAMIC heuristic genetic algorithm and the greedy-based approximation algorithm. The experimental results are shown in Figure 11 and Figure 12.

It can be seen that the compression ratio based on the heuristic genetic algorithm has obvious advantages in different size sample sets compared with an approximation algorithm. With the increase in sample size, the test time of the heuristic genetic algorithm is more and more advanced.

## 5. Discussion on Security and Privacy of CVDF DYNAMIC Model

Because CVDF DYNAMIC combines the bi-LSTM neural network and the genetic algorithm to generate fuzzy testing samples, the final sample set is a mixed sample set, and the sample set has no label for classification. Therefore, it is very difficult to deduce the sensitive training data of CVDF DYNAMIC through the final sample set generated by CVDF DYNAMIC. On the other hand, in the description of experiment part 4.1, the training data of CVDF DYNAMIC comes from the vulnerability databases of many different countries or companies. Some of these databases are open access and some are private, but CVDF DYNAMIC adopts mixed training for datasets from different sources in the training process and randomly selects 80% as the training set and 20% as the testing set in the mixed datasets. Therefore, even if the attacker obtains the CVDF DYNAMIC datasets through reverse derivation, it is also very difficult to further distinguish private data from the middle. However, the bi-LSTM neural network adopted by CVDF DYNAMIC is a mature neural network structure, and there are corresponding scientific studies to attack this neural network structure. The security of the bi-LSTM neural network structure still needs to be strengthened in the future.

## 6. Conclusions

Existing fuzzy testing tools and methods only focus on the code coverage or the test case generation on the critical path. It is difficult to take both the code coverage and path depth detection ability into account. Therefore, this paper proposes CVDF DYNAMIC, a fuzzy testing sample generation framework based on the bi-LSTM and the genetic algorithm.

By combining the genetic algorithm and the bi-LSTM neural network, the framework has the ability of code coverage and path depth detection and has excellent comprehensive performance. This paper also proposes path depth detection ability, which is an evaluation metrics of vulnerability detection ability under critical execution path. Meanwhile, a heuristic genetic algorithm is used for simplifying the sample set. Finally, the experimental results show that CVDF DYNAMIC is feasible and effective, and its performance is improved compared with existing fuzzy testing tools, such as AFLFast and NeuFuzz in several evaluation indices. (FPR, TPR, ACC, Code Coverage and WDC). The reduction in the sample set further improves the efficiency of the CVDF DYNAMIC test case generation. In the future, we will further optimize the performance of CVDF DYNAMIC by optimizing the neural network structure in CVDF DYNAMIC and perfecting the iterative rules of genetic algorithm and integrate more fuzzy testing sample generation methods to further improve the code coverage and path depth detection ability.

## Figures and Tables

**Table 1:** Dataset Information of CVDF DYNAMIC.

Data Sources	Types of Vulnerabilities
SARD	(CWE-119, CWE-120, CWE-131, CWE-134 etc)
Security Focus	(CWE-119, CWE-120, CWE-189, CWE-369 etc)
Github	(CWE-415, CWE-476, CWE-119, CWE-763 etc)

**Table 2:** Comparison Test Results Of CVDF DYNAMIC With Other Tools.

	Evaluation Indicator	FPR	TPR	ACC	Code Coverage	WDC
Tool						
CVDF DYNAMIC		5.6%	92.3%	88.9%	89.6%	2.76
NeuFuzz		10.2%	79.8%	83.4%	24.7%	2.78
VDiscover		8.5%	86.7%	85.8%	86.5%	2.33
AFLFast		11.2%	88.7%	82.9%	80.1%	1.94

**Table 3:** Index Comparison Of Sample Set Before And After Compression.

	Number of Samples	Compression Ratio	Execution Times/s	Code Coverage	WDC
Initial sample set	6308	54.6%	46,184	89.6%	2.76
Compressed sample set	2864		32,428	89.6%	2.76

## Data Availability

Data available on request due to restrictions eg privacy or ethical. The data presented in this study are available on request from the corresponding author. The data are not publicly available due to [Data privacy issues].
